# Crosstalk between ubiquitination and translation in neurodevelopmental disorders

**DOI:** 10.3389/fnmol.2024.1398048

**Published:** 2024-09-02

**Authors:** Nagore Elu, Srividya Subash, Susana R. Louros

**Affiliations:** ^1^Centre for Discovery Brain Sciences, University of Edinburgh, Edinburgh, United Kingdom; ^2^Simons Initiative for the Developing Brain, Patrick Wild Centre, University of Edinburgh, Edinburgh, United Kingdom

**Keywords:** ubiquitin, translation, splicing, ribosome, neurodevelopmental disorders, FMRP, UBE3A

## Abstract

Ubiquitination is one of the most conserved post-translational modifications and together with mRNA translation contributes to cellular protein homeostasis (proteostasis). Temporal and spatial regulation of proteostasis is particularly important during synaptic plasticity, when translation of specific mRNAs requires tight regulation. Mutations in genes encoding regulators of mRNA translation and in ubiquitin ligases have been associated with several neurodevelopmental disorders. RNA metabolism and translation are regulated by RNA-binding proteins, critical for the spatial and temporal control of translation in neurons. Several ubiquitin ligases also regulate RNA-dependent mechanisms in neurons, with numerous ubiquitination events described in splicing factors and ribosomal proteins. Here we will explore how ubiquitination regulates translation in neurons, from RNA biogenesis to alternative splicing and how dysregulation of ubiquitin signaling can be the underlying cause of pathology in neurodevelopmental disorders, such as Fragile X syndrome. Finally we propose that targeting ubiquitin signaling is an attractive novel therapeutic strategy for neurodevelopmental disorders where mRNA translation and ubiquitin signaling are disrupted.

## Introduction

Protein abundance is regulated by the coordination of synthesis and degradation, and these two processes sculpt the molecular architecture of a neuron during development and plasticity. The fate of proteins within cells is directly regulated by ubiquitination. This post-translational modification involves the covalent attachment of a small protein, ubiquitin, to target proteins. Ubiquitination is a cascade of events that start with the activation of ubiquitin by the E1 activating enzyme. Active ubiquitin is then transferred to the E2 conjugating enzyme, which is in charge of interacting with the E3 ligase to ultimately transfer ubiquitin into E3 ligase substrates. Mono-ubiquitination of proteins can regulate cellular processes such as gene transcription, signal transduction and DNA damage response by altering protein localization, protein-protein interactions or endocytosis (Greer et al., 2003; Pelzer et al., 2013; Fukushima et al., 2015; Zhou et al., 2017; Wang et al., 2019). In addition, ubiquitin can generate different types of chains via its seven lysine residues, and each type of poly-ubiquitination (N1, K6, K11, K27, K29, K33, K48, and K63) will have a distinct intracellular role. For instance, the K48 chain-type has been widely described to target proteins into degradation by the Ubiquitin Proteasome System (UPS; Thrower et al., 2000), while K63 ubiquitin chains can drive changes in protein localization or regulate endocytosis and innate immune response (Richard et al., 2020; Madiraju et al., 2022; Saeed et al., 2023).

Ubiquitin signaling has been widely described to be involved in receptor trafficking, synapse formation (Haas and Broadie, 2008; Iwai, 2021; Lei et al., 2021; Pérez-Villegas et al., 2022; Dikic and Schulman, 2023) and remodeling (Mei et al., 2020) by regulating the turnover of synaptic proteins at synapses (Tai and Schuman, 2008; Bingol and Sheng, 2011). Disruption of any ubiquitin-mediated pathways leads to aberrant neuronal morphology, connectivity or synapse formation, which are hallmark features of neurodevelopmental disorders (NDDs; Louros and Osterweil, 2016; Batool et al., 2019).

Neurodevelopmental disorders affect more than 15% of children worldwide (Romero-Ayuso, 2021), and include intellectual disability (ID) and autism spectrum disorder (ASD; Ismail and Shapiro, 2019). Large-scale sequencing studies contributed to the understanding of NDDs pathophysiology by unveiling their genetic etiology. More precisely, these studies report mutations in genes involved in synaptic function and structure, transcriptional and translational regulators (De Rubeis et al., 2014; Krumm et al., 2014; de la Torre-Ubieta et al., 2016), as well as mutations in genes involved in ubiquitin-dependent protein degradation (Louros and Osterweil, 2016; Trost et al., 2022). Several components of the UPS implicated in NDDs, also play crucial roles in RNA synthesis and splicing (Cho et al., 2014; Jewell et al., 2015; Saez et al., 2020; Pitts et al., 2022). Therefore, there is a growing interest in exploring the crosstalk between protein synthesis and protein degradation in the context of NDDs. This mini-review aims to compile all pertinent information on the regulatory role of ubiquitination in RNA biogenesis in the context of NDDs.

## Ubiquitin ligases as new players in translation control

Neurons maintain efficient crosstalk between protein translation and degradation to adjust to their physiological needs. Among the intermediaries between protein synthesis and degradation pathways, ubiquitin ligases emerge as central regulators. Ubiquitination of ribosomal proteins has been described over three decades in a seminal paper showing that ubiquitination regulates ribosomal proteins abundance and that the assembly into the ribosome is facilitated by ubiquitin (Finley et al., 1989). More recent studies showed that K63-linked ubiquitination of ribosomal proteins and translation elongation factors promote translation in yeast (Silva et al., 2015). Similarly, in human cells ribosomal proteins were found to be ubiquitinated after the inhibition of translation (Higgins et al., 2015). Moreover, Culin-3, an E3 ubiquitin ligase previously implicated in NDDs (De Rubeis et al., 2014), has been involved in the formation of a ribosome modification platform that alters the translation of specific mRNAs (Werner et al., 2015). Ubiquitination can also regulate translation is by modulating translational surveillance pathways. When aberrant nascent polypeptides are stalled in ribosomes during translation and ribosomes collide, the ribosome quality control (RQC) surveillance pathway is activated, in which ubiquitinated ribosomal subunits are recognized to assist into the ribosome-splitting event (Matsuo et al., 2023). Although dysfunction of RQC is suggested to elicit neurological disorders, the molecular mechanisms involved remain poorly understood. Makorin ring finger protein (MRKN1), a ubiquitin ligase previously shown to control local translation in neurons during synaptic plasticity (Miroci et al., 2012), was recently implicated in the RQC pathway, promoting ribosome stalling at poly(A) sequences and starting RQC by ubiquitinating RPS10 and other RQC factors (Hildebrandt et al., 2019). Interestingly, MRKN1 is member of a family of ubiquitin ligases that also binds RNA, known as the RNA-binding ubiquitin ligases (RBULs). So far, over 30 RBULs have been identified (Thapa et al., 2020) but their function in the brain remains elusive.

Although previous studies demonstrate a direct link between ribosomal protein ubiquitination and changes in translation, the role of ribosomal protein ubiquitination in neurons hasn't been explored. The majority of ribosomal proteins is produced in the nucleus where ribosomes are assembled, but the enrichment of mRNAs of ribosomal proteins in dendrites and axons is a long-standing observation (Moccia et al., 2003). Proteomic studies show that over 80% of ribosomal proteins are ubiquitinated in neurons (Schreiber et al., 2015; Sun et al., 2023), 20 of those putatively ubiquitinated in synaptic fractions (Na et al., 2012; Table 1). Recent studies confirmed that ribosomal proteins are locally synthesized and incorporated into existing ribosomes in axons (Shigeoka et al., 2019) as well as in dendrites (Fusco et al., 2021). Both studies show that a subset of ribosomal proteins is more frequently incorporated or exchanged into mature ribosomes. Interestingly, a fraction of the exchanging ribosomal proteins is also ubiquitinated in neurons (Table 1; Na et al., 2012; Schreiber et al., 2015), suggesting an additional layer of regulation of ribosomal protein exchange in neurons that may be essential to regulate local protein synthesis in response to synaptic plasticity. Whether these processes are affected in NDDs is an open question, but since changes in ribosome abundance have been reported in several NDDs (Griesi-Oliveira et al., 2020; Seo et al., 2022), it would be interesting to investigate if their ubiquitination is aberrant in NDDs, and if that can be targeted to normalize ribosome levels and translation rates.

**Table 1:** Several components of the splicing machinery as well as ribosomal proteins are ubiquitinated in the brain.

**Cellular compartment**	**Protein names**	**References**
Ribosome	RPL11, RPL12^*^, RPL13, RPL13A, RPL14^*^, RPL15, RPL17, RPL18, RPL18A, RPL24, RPL26, RPL27, RPL27A, RPL3, RPL32, RPL34, RPL35, RPL39, RPL4, RPL5, RPL6, RPL7, RPL7L1, RPL8^*^, RPL9, RPS10, RPS11, RPS14, RPS15, RPS15A, RPS16, RPS19^*^, RPS2, RPS23, RPS25^*^, RPS27, RPS27A, RPS29, RPS3^*^, RPS3A, RPS5, RPS6, RPS7, RPS8, RPS9	Sun et al., 2023
	RACK1^*^, RPL19, RPL23A, RPL30^*^, RPS20, RPS21, RPSA	Na et al., 2012
	RPL10A, RPL28^*^, RPL29, RPL31, RPL35A, RPL38^*^, RPL7A, RPS13^*^, RPS17, RPS18^*^, RPS24, RPS26, RPS4X	Schreiber et al., 2015
Spliceosome	CDC5L, DDX46, EIF4A3, LUC7L3, PRPF19, PRPF3, PRPF8, RPL11, RPL18A, RPL39, RPS23, RPS29, SF3A3, SF3B6, SMU1, SNRNP200, SNRNP70, SNRPA1, SNRPB2, SNRPF	Sun et al., 2023
	CWC22, CWC27, DDX23, IK, RBMXL2, SF3B1, SNRNP35, SNRPD3, SNRPE, SNRPN, SRRM2, USP39, YBX1	Schreiber et al., 2015

^*^Ubiquitination events found in synaptic fractions.

## Alternative splicing regulation by ubiquitin and its dysfunction in NDDs

Most protein-coding genes in humans are transcribed as pre-mRNAs that contain a series of exons and introns. Following transcription, the removal of introns during the process of pre-mRNA splicing is required before the nascent transcript is translated into a protein. Alternative splicing generates multiple proteins from a single pre-mRNA by including and/or excluding alternative exons, thereby diversifying cellular proteomes (Han et al., 2011; Wang et al., 2015). This process is particularly important in neurons that rely on the function of heavily spliced genes such as Neurexins, n-Cadherins, and calcium-activated potassium channels that can produce hundreds of mRNA isoforms through alternative splicing. Indeed, some NDDs occur when alternative splicing goes awry. For example, extensive transcriptomics studies using post-mortem brain tissue from ASD patients have shown pervasive mis-regulation of microexon splicing (Irimia et al., 2014; Chanarat and Mishra, 2018; Su et al., 2018).

The molecular machinery responsible for pre-mRNA splicing is called the spliceosome complex. It is composed of five small nuclear RNAs (U1, U2, U4, U5, and U6) pre-assembled with proteins into small ribonucleoproteins (snRNPs), together with hundreds of auxiliary proteins that help the spliceosome recognize splice sites (Wassarman and Steitz, 1992; Zhou et al., 2002; Matlin and Moore, 2007). High-throughput genetic studies showed a possible link between ubiquitin ligases and the process of pre-mRNA splicing. For instance, ubiquitin binds to the highly conserved spliceosomal core protein PRPF8 via its C-terminal domain (Grainger and Beggs, 2005; Bellare et al., 2006, 2008). Additionally, the literature also suggests that ubiquitination of other splicing factors may modulate spliceosomal activity through reversible protein-protein interactions (Bellare et al., 2008). For example, PRPF3 and PRPF31 undergo K63-linked ubiquitination by an RBUL, PRPF19 (Chanarat and Mishra, 2018), an essential step for spliceosomal activation (Hogg et al., 2010). Ubiquitinated PRPF3 and PRPF31 then bind PRPF8 and stabilize the tri-snRNP complex (Park et al., 2016). As the splicing cycle progresses, PRPF3 and PRPF31 are deubiquitinated by USP4 and USP15, respectively (Song et al., 2010; Das et al., 2017). Altogether, this shows that the ubiquitination state of several components of the spliceosome tightly regulate its assembly and activation, therefore affecting splicing.

The regulation of the spliceosome by ubiquitination in neurons is less elucidated, but proteomic studies identify several ubiquitinated splicing factors such as PRPF3, PRP9, as well as the RBUL, PRPF19 (Table 1). Considering that neurons express highly spliced genes, dysregulated ubiquitination of the spliceosome could have major consequences in neuronal development and function and contribute to NDDs. Importantly, a recent study identified mutations in three spliceosome factors in NDDs, including six individuals who harbored mostly *de novo* heterozygous variants in *PRPF19*. This study demonstrated that these pathogenic variants lead to converging neurodevelopmental phenotypes, including, but not limited to developmental delay, ID and autism (Li et al., 2024).

## Ubiquitination of RNA-binding proteins: contribution to NDDs

RNA metabolism is regulated at different stages by specific RNA-binding proteins (RBPs). RBPs are responsible for mRNA transport and translation regulation within dendrites and are required for long-lasting forms of synaptic plasticity (Glock et al., 2017). The loss of RBP function leads to numerous disorders, including ASD, Fragile X Syndrome (FXS; Bhakar et al., 2012; Zoghbi and Bear, 2012; Darnell and Klann, 2013; Lee et al., 2016; Popovitchenko et al., 2016) and epilepsy (Lee et al., 2016).

Due to its significant role in translation regulation and its impact on neuronal homeostasis, Fragile X messenger ribonucleoprotein (FMRP) stands out as one of the most extensively studied RBPs. Evidence suggests that FMRP is transported into dendrites and synapses where it acts as a central regulator of local translation (Darnell and Klann, 2013; Schieweck et al., 2021). Additionally, FMRP has a dual role in both RNA localization and translation; localizes to polyribosome complexes and is well-documented for its role as a translational repressor (Laggerbauer et al., 2001; Mazroui et al., 2002). Studies of *Fmr1* mutant models have revealed alterations in plasticity and excitability in several brain circuits, as a consequence of the excessive protein synthesis (Osterweil et al., 2013; Louros et al., 2023). Deficiency of FMRP, the underlying cause of Fragile X Syndrome, causes dysregulation of the translation of mRNAs that bind to FMRP. Interestingly, the majority of FMRP target mRNAs are less translated in the hippocampus (Ceolin et al., 2017; Thomson et al., 2017; Sawicka et al., 2019; Sharma et al., 2019; Seo et al., 2022) and this is reflected in the synapse-enriched proteome of *Fmr1* KO mouse (Louros et al., 2023).

FMRP undergoes degradation primarily through the ubiquitin-proteasome system (UPS), which is a major pathway for targeted protein degradation in cells (Chanarat and Mishra, 2018; Ebstein et al., 2021; Winden et al., 2023). Consistent with this, FMRP undergoes regulation by ubiquitination, a tightly controlled process that can be triggered by specific events such as dephosphorylation at key sites such as S499 (Wilkerson et al., 2023). Various factors contribute to this dephosphorylation, including activation of PP2A by the activation of metabotropic glutamate receptors (Nalavadi et al., 2012). Additionally, developmental cues play a crucial role during specific stages of development by regulating dephosphorylation and subsequent ubiquitination of FMRP (Schieweck et al., 2021). Once dephosphorylated, FMRP becomes a target for specific E3 ubiquitin ligases such as APC/Cdh1, and once ubiquitinated, it is targeted for degradation (Nalavadi et al., 2012; Valdez-Sinon et al., 2020). FMRP is also known for its role in mRNA-protein interactions within ribonucleoprotein (RNP) granules, which are crucial for mRNA transport and localization (Valdez-Sinon et al., 2020). Ubiquitination-induced degradation of FMRP may disrupt these interactions, impairing the transport and proper localization of mRNAs, thereby affecting gene expression programs that are essential for normal cellular function (Valdez-Sinon et al., 2020; Wilkerson et al., 2023).

In addition to FMRP other RBPs related to NDDs are regulated by ubiquitination. One example is the ELAVL family, which undertakes essential functions across spatiotemporal windows in brain development to help regulate and specify transcriptomic programs for cell specialization (Mulligan and Bicknell, 2023). Different components of this family have been related to ASDs, behavioral abnormalities or seizures (Mulligan and Bicknell, 2023), with ELAV2 showing a clear role in neurodevelopment and listed by SFARI as a candidate gene for ASD. ELAV2 targets are also involved in synaptic function and neurodevelopmental disorders (Berto et al., 2016). Even though in the context of cancer, ELAV1 has been described to be ubiquitinated, facilitating its proteasome mediated degradation and leading to an increase in the survival rate of cells under heat-shock response (Daks et al., 2021), the ubiquitination of this RBP has not been demonstrated in neurons. RBFOX1 is another RBP strongly implicated in ASD. This protein regulates both splicing and transcriptional networks in human neuronal development (Fogel et al., 2012) and it has been found to be ubiquitinated in Alzheimer's disease post mortem human brain tissue, particularly in axons, tangles and neuropil threads, suggesting a role in axonal proteostasis (Fernandez et al., 2021).

Altogether, these evidences show that RNA binding proteins are regulated by ubiquitination in the brain and despite lacking detailed mechanisms it is possible that aberrant ubiquitination of RNA binding proteins contributes to the pathophysiology of NDDs.

## Dysregulated proteostasis in NDDs: new therapeutic opportunities

Dysregulation of translational represents a common endpoint of familial and sporadic ASD-associated signaling pathways (De Rubeis et al., 2014; Krumm et al., 2014). The identification of this dysregulated pathway has been used to develop several therapeutic strategies for FXS and other NDDs, however, due to the limited success in clinical trials there is an urgent need for identification of new pathways amenable for therapeutic development.

A recent development was the discovery of upregulated protein degradation machinery in FXS, downstream of the increased protein translation rates that characterize this disorder (Louros et al., 2023). This study shows that the increase in protein degradation is primarily a consequence of excessive translation of proteasomal subunits and ubiquitin ligases in excitatory neurons from *Fmr1* mutant mice. Importantly, pharmacological reduction of proteasome activity and ubiquitin ligases was sufficient to normalize protein synthesis rates, demonstrating the intricate relationship between translation and degradation in FXS. This could be a consequence of modulating ribosomal subunits turnover since the authors found ribosomal subunits excessively targeted for degradation in synaptic enriched fractions, possibly through increased ubiquitination rates. Finally, this study found that increased proteasome activity contributes to hyperexcitability and audiogenic seizures in Fmr1 KO mice, and that this phenotype was corrected by pharmacological and genetic manipulation of the proteasome (Louros et al., 2023). This study opens the door to more investigations into the dysfunction of ubiquitin signaling and proteasomal degradation in other NDDs, and it demonstrates that targeting ubiquitin signaling could be a new pathway for therapeutic development.

One of the most studied ubiquitin ligases linked to neurodevelopmental disorders is UBE3A, with loss of function mutations causing Angelman syndrome (AS; Kalsner and Chamberlain, 2015). AS is characterized by intellectual disability, developmental delay, seizures, motor disruptions, and an unusually positive demeanor (LaSalle et al., 2015). Many studies have identified targets of Ube3a in mouse, rats and human AS samples (Pandya et al., 2022) including some regulators of protein synthesis. One interesting target of Ube3a is the mTOR suppressor protein TSC2 (Zheng et al., 2008), directly involved in the regulation of protein synthesis. Recent work suggests that degradation of TSC2 following ubiquitination by Ube3a may contribute to pathology, as treatment with the mTOR inhibitor rapamycin rescued motor deficits and abnormal dendritic branching in AS mutant mice (Sun et al., 2015). Furthermore, lovastatin, previously shown to correct excessive protein synthesis rates and seizures in FXS (Osterweil et al., 2013; Asiminas et al., 2019), was also shown to correct seizures in the AS mouse model (Chung et al., 2018), suggesting that protein synthesis rates could be increased in the AS mutant mouse. This was indeed confirmed in a recent study that found increased *de novo* protein synthesis in the hippocampus of the AS mutant mouse (Aria et al., 2023), as well as impaired autophagy that when enhanced was able to ameliorate cognitive impairments in AS mice.

Altogether, these findings show the intricate crosstalk between ubiquitin signaling and translation in NDDs. Targeted protein degradation technologies have emerged over 20 years ago with potential for targeting undruggable protein targets. PROTAC (proteolysis-targeting chimera) or molecular glue (MG)-driven ternary complex formation with an ubiquitin E3 ligase utilizes cells' UPS to degrade target proteins. Several such molecules have entered clinical development (Kong and Jones, 2023). Recent clinical proof-of-concept for PROTAC molecules against two cancer targets confirmed the successful clinical targeting of proteins previously considered “undruggable.” There are currently over 20 new PROTACs under clinical development (Békés et al., 2022). The application of these strategies to brain disorders offers several advantages and challenges but recent studies have shown promise in the context of neurodegenerative disorders [recently reviewed by Farrell and Jarome (2021)] suggesting that this new therapeutic avenue for NDDs could offer increased specificity and lower off-target effects.

## Conclusions and perspectives

**Figure 1:**
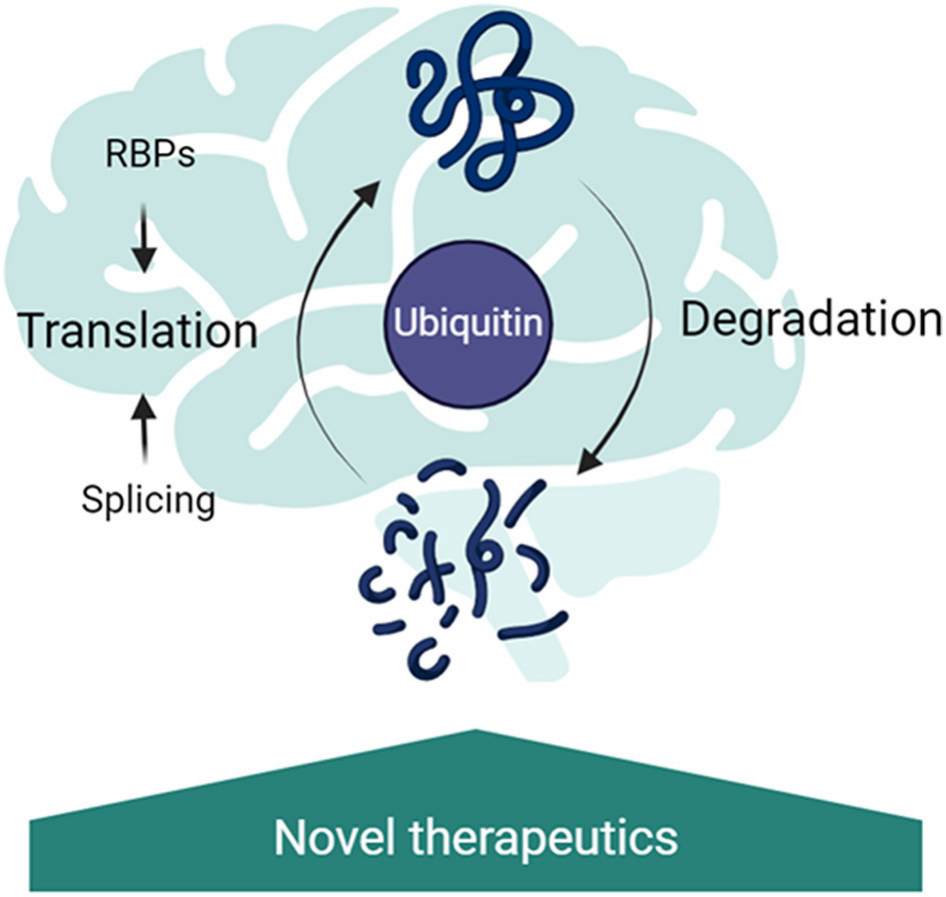
Ubiquitin signaling as a central regulator of RNA metabolism in the brain. Ubiquitination regulates splicing, through regulation of the spliceosome remodeling; RNA-binding protein abundance and binding partners; translation rates through ribosomal protein ubiquitination and the turnover rates of proteins in the brain. Disruption of these pathways has been implicated in neurodevelopmental disorders, therefore targeting the ubiquitin signaling is a promising new therapeutic strategy.

Molecular analysis of patient-derived tissues and mouse models of the monogenic ID has shown widespread changes at the epigenetic, transcriptional, and translational gene expression levels. The interplay between changes at multiple levels is essential to the pathophysiology of NDDs. Importantly, coordination between the translational machinery, RBPs and the ubiquitin proteasome system regulates dendritic proteostasis in response to neuronal activity (Hanus and Schuman, 2013). Indeed, mutations in components of these systems are associated with altered plasticity and may underlie the pathogenesis of NDDs. Considering that in several models of NDDs protein synthesis rates are affected (Auerbach et al., 2011; Barnes et al., 2015; Aria et al., 2023), ribosome abundance is increased and the ubiquitin proteasome system is overexpressed (Seo et al., 2022; Louros et al., 2023), it is pertinent to investigate the contributions of ubiquitin signaling dysfunction to ribosome quality control and alternative splicing. However, the isolation and identification of ubiquitinated proteins under physiological conditions from *in vivo* tissues is a challenging task, particularly in the brain, as the ubiquitinated proteins are generally found at very low levels within the cells. Besides, the fast kinetics at which some of the proteins conjugated with ubiquitin are degraded (Ronchi and Haas, 2012), the action of the deubiquitinating enzymes (Stegmeier et al., 2007) or the fact that proteins might be modified with ubiquitin only in well-defined temporal windows (Clute and Pines, 1999), make their analysis even more challenging. Considering that ubiquitin signaling modulates so many aspects of RNA biogenesis that are affected in NDDs (Figure 1), we believe it is vital to develop methods to improve the identification of dysregulated ubiquitination in the brain to accelerate the development of novel therapeutic options for NDDs.
